# Cohort Profile Update: Finnish Health in Teens (Fin-HIT)

**DOI:** 10.1093/ije/dyaf025

**Published:** 2025-03-25

**Authors:** Catharina Sarkkola, Sohvi Lommi, Emilia Ankkuri, Dylan Pashley, Kris Elomaa, Nitin Agrawal, Elina Engberg, Satu Männistö, Jari Lahti, Heli Viljakainen

**Affiliations:** Folkhälsan Research Center, 00250 Helsinki, Finland; Department of Public Health, Faculty of Medicine, University of Helsinki, 00290 Helsinki, Finland; Folkhälsan Research Center, 00250 Helsinki, Finland; Faculty of Medicine, University of Helsinki, 00290 Helsinki, Finland; Folkhälsan Research Center, 00250 Helsinki, Finland; Faculty of Medicine, University of Helsinki, 00290 Helsinki, Finland; Folkhälsan Research Center, 00250 Helsinki, Finland; Folkhälsan Research Center, 00250 Helsinki, Finland; Folkhälsan Research Center, 00250 Helsinki, Finland; Faculty of Medicine, University of Helsinki, 00290 Helsinki, Finland; Folkhälsan Research Center, 00250 Helsinki, Finland; Department of Psychology and Logopedics, Faculty of Medicine, University of Helsinki, 00290 Helsinki, Finland; Finnish Institute for Health and Welfare (THL), 00300 Helsinki, Finland; Department of Psychology and Logopedics, Faculty of Medicine, University of Helsinki, 00290 Helsinki, Finland; Folkhälsan Research Center, 00250 Helsinki, Finland; Faculty of Medicine, University of Helsinki, 00290 Helsinki, Finland

**Keywords:** prospective cohort study, young adults, weight status, overweight, obesity, diet, mental health, substance use, biobank, Finland

Key Features

- The Finnish Health in Teens study is a school-based cohort study of initially 11 407 11-year-old adolescents that was established in 2011. The cohort was set up to investigate environmental and genetic determinants of weight development from early adolescence to adulthood.
- The third data collection was conducted when the participants reached adulthood (hereafter called ‘young adults’). The main research areas were diet and mental health. Moreover, consent to store collected research data in the Finnish Institute for Health and Welfare (THL) Biobank was requested.
- In total, 3935 young adults with a mean (standard deviation) age of 19.5 (0.9) years participated in the third wave in 2020–2024.
- The web survey included validated questionnaires on habitual diet, disordered eating, symptoms of depression and anxiety, perceived stress, insomnia, and smartphone addiction.
- Requests for collaboration and data sharing can be sent to Principal Investigator Heli Viljakainen (heli.viljakainen@helsinki.fi). Selected variables can be accessed through the THL Biobank and the European Genome-phenome Archive.

## The original cohort

**Figure 1.**
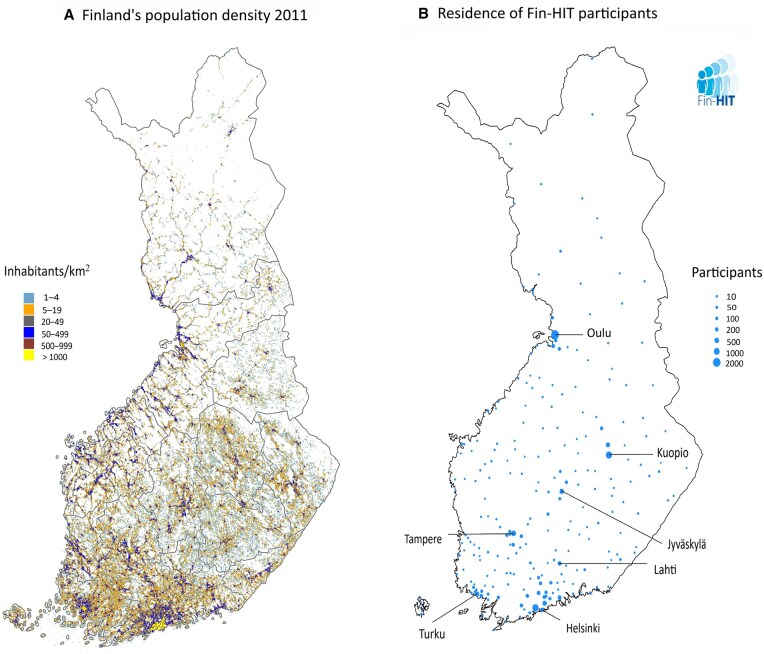
Population density of Finland in 2011 (A) and recruitment of Finnish Health in Teens (Fin-HIT) study participants in 2011–2014 (B). This figure was first published in the doctoral dissertation by Sohvi Lommi [42]. Written permission to reproduce the figure was received from the copyright owner.

The Finnish Health in Teens (Fin-HIT) study was initiated in 2011 to investigate environmental and genetic factors associated with weight and weight gain from early adolescence to adulthood [1]. The baseline cohort (first wave) included 11 407 adolescents aged 9–12 years and 9935 parents, of whom 86.8% were mothers. Recruitment was mainly carried out in schools in Finland’s largest cities and their surroundings (Figure 1). Of all the invited adolescents, including the pilot and main study combined, 30% enrolled in the study (Supplementary Figure S1).

At the first wave, the adolescents’ height, weight, and waist circumference were measured at school by trained fieldworkers. In addition, the participants self-reported their lifestyle factors in an online survey and gave a saliva sample (Oragene^®^ DNA Self-Collection Kit, DNA Genotek Inc., Canada) (as explained elsewhere) [1]. The parental questionnaire included background information on the family, the adolescent’s early-life events and eating behaviors, and the parent’s anthropometry, and was available from 6046 parents.

The first-wave participants were reinvited in 2015 for the second wave of data collection. The adolescents were 14 years old on average and the participation rate was 53.5% (*n* = 5911) (Supplementary Figure S1). The participants self-reported their anthropometric and questionnaire data. We have previously demonstrated the feasibility and accuracy of home-based anthropometric measurements for epidemiological studies [2]. In addition, the adolescent and the parent mailed self-collected saliva samples, which have been used for omics analysis.

The current ethical approval allows a 25-year-long follow-up period, which all participants have consented to. It includes linkage to national health registers with excellent coverage [3], such as the Medical Birth Register, Register of Primary Health Care visits, and Care Register for Health Care held by the Finnish Institute for Health and Welfare (THL), as well as the Drug Purchases Register and Special Reimbursement Register maintained by the Social Insurance Institution of Finland. The register data provide a comprehensive view of the participants’ health status, as well as their weight trajectories across the school-age years based on school health examinations.

## What is the reason for the new data collection?

This prospective cohort study aims to follow changes in environmental exposures, behavioral factors, weight, and overall health over time, and investigate long-term and temporal associations between the measures. Thus, the new data collection advances the aims of the study by following up on the participants as they reach the brink of adulthood. Between ages 18 and 25 years, weight gain and incidence of overweight are greater than during any other period of life [4–6].

New or accumulated behavioral risk factors for weight gain and overweight may appear in emerging adulthood [4]. These factors may include unfavorable eating habits [4], lower leisure-time physical activity, substance abuse, and discrepancy in daily schedules between weekdays and weekends. During childhood and adolescence, sedentary digital media use increases gradually [5, 6], which may disturb sleep, impair concentration skills, and decline academic performance [7]. Some individuals are prone to developing behavioral addiction to smartphone use [8].

Emerging adulthood is characterized by unique features that distinguish it from extended adolescence or adulthood [9]. This stage is defined by identity exploration, instability, a focus on developing oneself, a feeling of being in between childhood and adulthood, and exploration of life possibilities [9]. It encompasses several milestones in life: transitions in school, work, and family life, as well as in romantic relationships, leading to changes in social relationships, gaining independence, and navigating stress and emotions. At the same time, young adults must assume responsibility for their health and lifestyle choices, which, in today’s obesogenic society, is demanding. Emerging adulthood is also a period when mental disorders, particularly mood disorders, manifest [10]. New data are warranted to understand the contributing factors in the transition phase and interconnections of mental health with general health and wellbeing.

Finnish biobanks administrate large sample collections, which are of great importance in research on the Finnish population’s health. Therefore, all Fin-HIT participants were asked for their consent to deposit their saliva samples and research data in the THL Biobank (https://www.thl.fi/biobank) during the third wave. Biobanking enables more extensive use of our data, such as developing new treatment methods and preventing diseases. For example, in the framework of a Finnish genomics research project called FinnGen (https://www.finngen.fi/en), the Fin-HIT samples that were deposited in the biobank were extracted, genotyped, and imputed with the Finnish-specific SISu v3 reference panel (https://thl.fi/en/research-and-development/thl-biobank/for-researchers/sample-collections/thl-biobank-imputation-reference-panel), which tremendously increased the number of available genotypes for future studies.

## What will be the new areas of research?

The incidence of mental health disorders increases from the teenage years onwards, with nearly two-thirds of the onsets of first mental disorder occurring before the age of 25 years [10]. For example, in Finland, ∼30% of university students have experienced sleep problems, depression, or burnout [11]. Similar numbers have been reported in other European countries [12–14]. Understanding the underpinnings of young adults’ mental health as well as the consequences of poor mental health is crucial, as these challenges can affect their subsequent physical, psychological, and social wellbeing, impacting their future opportunities and overall health and life satisfaction [7]. On the other hand, substance abuse, smartphone addiction, and sleep problems are increasingly impacting mental and physical health [7]. We have assessed psychiatric symptoms, symptoms of depression and anxiety, perceived stress, sleep problems, smartphone addiction, and substance use with validated questionnaires in the young adults (Table 1). These data allow us to investigate causes and consequences between lifestyle factors and mental health in emerging adulthood that have remained unsolved.

**Table 1.** New topics and validated measures included in third-wave data collection

Topic	Name of questionnaire (abbreviation)	Reference
Food intake	THL Food frequency questionnaire (FFQ)	Männistö 1996 [16], Paalanen 2006 [17], Kaartinen 2012 [18]
Psychiatric symptoms	Symptom Checklist 90 (SCL-90)	Derogatis 1973 [33]
Depression	Center for Epidemiologic Studies Depression Scale (CES-D)	Radloff 1977 [34]
Anxiety	Beck Anxiety Inventory (BAI)	Beck 1988 [35]
Perceived stress	Perceived Stress Scale (PSS-10)	Cohen 1983 [36]
Insomnia	Insomnia Severity Index (ISI)	Morin 2011 [37]
Self-esteem	Rosenberg Self-Esteem Scale (SES)	Rosenberg 1989 [38]
Smartphone addiction	Smartphone Addiction Scale, short version (SAS-SV)	Kwon 2013 [39]
Disordered eating	Eating Attitudes Test (EAT-26)	Garner 1982 [19]
Alcohol abuse	The Alcohol Use Disorders Identification Test—Concise (AUDIT-C)	Saunders 1993 [40]

As the transition to a more sustainable food system is ongoing and diets contribute through health, environment, culture, and economy to sustainable food systems [15], we need to understand the food consumption patterns of future working-age people. Within a few decades, they will be managing society and raising the next generation. For example, it is imperative to estimate the proportion of young adults who are adhering to the planetary health diet [15] and to examine nutrients with concerning intake levels. This information will serve the authorities in the risk assessment and planning of health promotion activities. Beyond high energy intake, high intake of ultra processed foods and low intake of fruits and vegetables are common concerns in emerging adulthood [4]. Young adults have self-reported their consumption of meals, diet, and food with a validated food frequency questionnaire [16–18] and eating-related attitudes, feelings, and behaviors with the Eating Attitudes Test [19] that will be used in future studies.

## Who is in the cohort?

The third-wave data collection was conducted between September 2020 and April 2024, when the participants had reached their majority, i.e. were ≥18 years old. Of the initial cohort of 11 407 participants, we decided to invite those from whom we had received at least one valid body mass index (BMI) measurement or saliva sample from the first or second wave (*n* = 11 109). Of these, 20 had died and 98 were lost due to either a secret address or an unknown address abroad. Thus, the final number of invited participants was 10 991 (Supplementary Figure S1).

**Figure 2.**
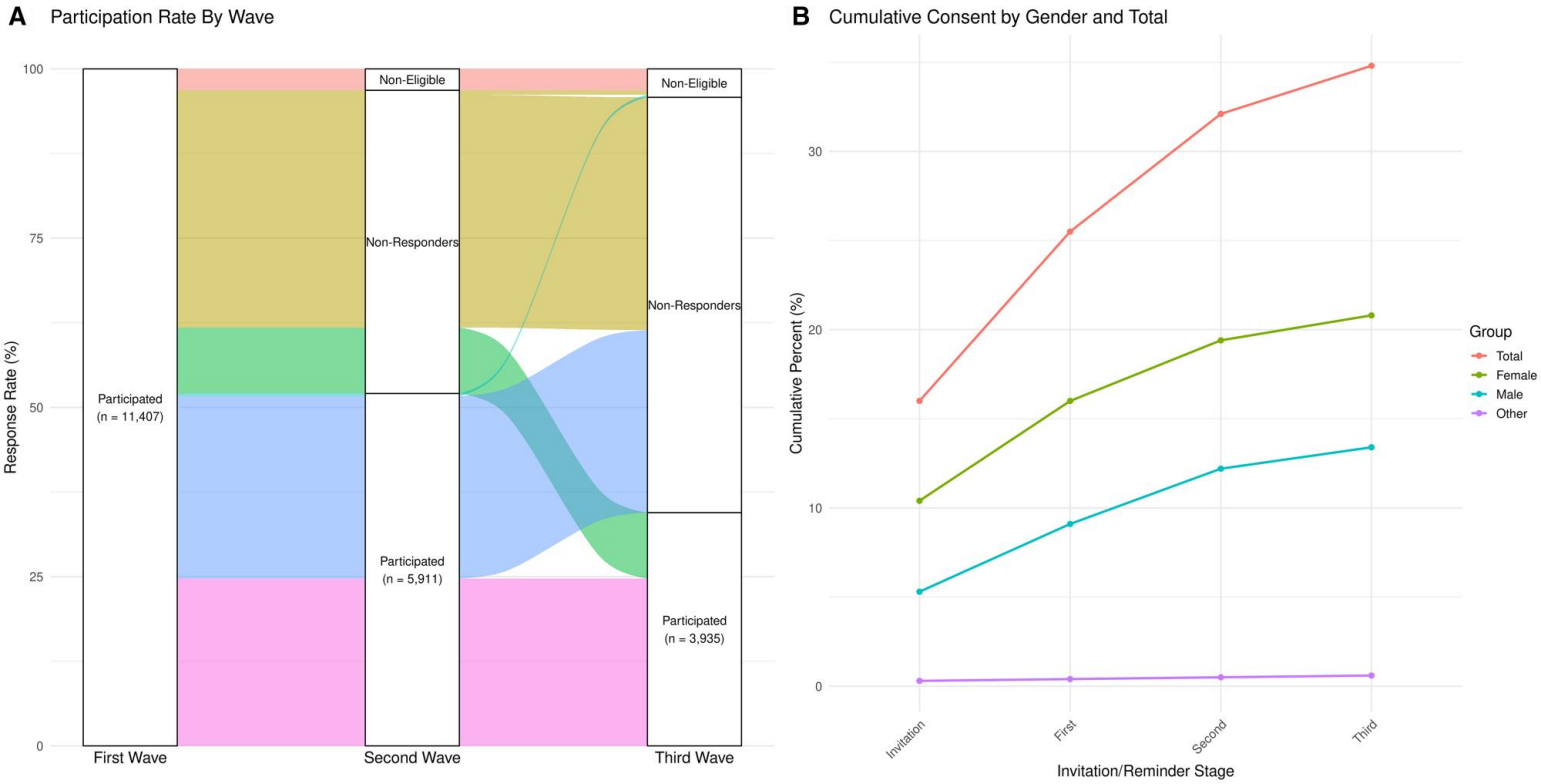
Participant retention and attrition across study waves and cumulative response rate after reminders in the third wave. (A) Alluvial plot illustrating participant retention and attrition across study waves. This figure presents an alluvial plot depicting the flow of participants through different waves of the study, described from top to bottom. • 1 (on top; red): Represents non-eligible participants, due to either missing data or withdrawal from the study. • 2 (yellow): Represents nonresponders in both the second and third waves. • 3 (green): Represents participants who were nonresponders in the second wave but responded in the third wave. • 4 (blue): Represents participants who responded in the second wave but did not participate in the third wave. • 5 (on bottom; lilac): Represents participants who participated in all three waves of the study. (B) Cumulative response rate after each round of reminders in the third wave of data collection for total, female, male, and other.

Invitation letters with personal login codes to the online consent form and questionnaire were sent by mail, which was the only option to reach the participants. The postal addresses were obtained from the Finnish Digital Agency. Non-respondents received two or three reminder letters at intervals of 3 and 5 weeks, and, additionally, most of the cohort at 6–8 months to boost participation (Figure 2B).

The participation rate for the third wave was 34.5% of the initial cohort (*n* = 3935 consented) with a mean (SD) follow-up time of 8.2 (0.9) years. Of those invited, 1.6% (*n* = 174) actively declined to participate in the current data collection, while 1.2% (*n* = 130) withdrew their consent from any current and future follow-ups of the study. Separate biobank consent on storing saliva samples and research data in the THL Biobank was obtained from 32.0% (*n* = 3522), while 4.8% (*n* = 523) declined storage of their data and samples in the biobank. In contrast to the first and second waves, no questionnaire was assigned to parents; only consent for the biobank was requested. Of the parents, 61.2% (*n* = 3051 out of 4988) consented to biobank data inclusion and 2.1% (*n* = 105) declined biobank data inclusion, while 0.8% (*n* = 39) withdrew their consent from any current and future participation in the study.

Of the participants, 60% were female (Table 2). The mean (SD) age of all third-wave participants was 19.5 (0.9) years, with an age range of 18–24 years. Sixty-six per cent reported that they were currently studying: the most common schools were upper secondary school (34.0%) and an institute of higher education or university (17.2%). In contrast, 11.4% reported having had a year off, 10.4% were working, and 7.8% were at military/nonmilitary service.

**Table 2.** Participant characteristics at first and third waves with *n* (%) if not indicated otherwise

	**First wave** (*n* = 11 407)		**Third wave** (*n* = 3935)	
Characteristic	*n*	%	*n*	%
Sex				
Female	5981	52.4	2350^a^	59.7
Male	5423	47.6	1530^a^	38.9
Other	–	–	55	1.4
Age, years^b^	11.2	(0.8)	19.5	(0.9)
BMI category^c^				
Thinness	1175	11.0	193	5.3
Normal weight	7854	73.8	2686	73.1
Overweight	1343	12.6	602	16.4
Obesity	274	2.6	191	5.2
(Missing values)			263	
Height, cm^b^^,^^d^	147.8	(8.6)	172.0	(9.5)
Waist circumference, cm^b^^,^^d^	64.2	(7.9)	78.3	(10.6)
Waist-to-height ratio (WtHR)^b^^,^^d^	0.43	(0.05)	0.45	(0.1)
Central obesity (WtHR ≥ 0.5)	961	(9.0)	601	16.4
(Missing values)			269	
Sex of parents who participated alongside their children				
Female	8568	86.8	1628	86.9
Male	1308	13.2	246	13.1
(Missing values)			18	
Maternal occupational status at birth				
Upper-level employees	3169	29.7	1291	36.2
Lower-level employees	4299	40.3	1347	37.8
Manual workers	1279	12.0	327	9.2
Students	1127	10.6	379	10.6
Others^e^	803	7.5	219	6.1
(Missing values)			372	
Current occupation^f^				
Studying			2467	66.0
Comprehensive school			10	0.3
Vocational school or similar			292	7.8
Technical institute or similar			33	0.9
Upper secondary school			1272	34.0
University of applied sciences (polytechnic)			216	5.8
Institute of higher education or university			644	17.2
Working			387	10.4
Having a year off			428	11.4
Military or nonmilitary service			293	7.8
On parental leave			7	0.2
Unemployed			94	2.5
Doing something else			62	1.7
(Missing values)			197	
Current habitation^g^				
Childhood home			2463	64.5
Alone			742	19.4
With a housemate or in a shared flat			249	6.5
With a spouse/partner			349	9.1
With a child/with children			13	0.3
(Missing values)			119	
Alcohol consumption				
Never			541	16.3
Monthly or less			1292	39.0
2–4 times a month			1287	38.8
2–3 times a week			178	5.4
4 or more times a week			16	0.5
(Missing values)			621	
Smoking				
Never			2552	77.1
Previous			254	7.7
Yes, sometimes			398	12.0
Yes, regularly			107	3.2
(Missing values)			624	
Smoke electronic cigarettes				
Never			3025	91.4
Previous			178	5.4
Yes, sometimes			95	2.9
Yes, regularly			13	0.4
(Missing values)			624	
Use snuff				
Never			2887	87.2
Previous			149	4.5
Yes, sometimes			111	3.4
Yes, regularly			164	5.0
(Missing values)			624	

a Self-reported gender, except for altogether 119 individuals without self-reports, whose sex was confirmed through the Population Information System.

b Presented with mean (SD).

c At first wave International Obesity Task Force [41].

d Missing values between 245 and 269.

e Self-employed, stay-at-home-mothers, unemployed, pensioners.

f Multiple schools could be selected; the highest level is reported here. For individuals in studies, other occupations were not asked for.

g Multiple options could be selected; the last is reported here.

The majority (64.5%) of participants lived in their childhood home; 19.4% lived alone, 9.1% lived with a partner, and 6.5% shared a flat with others. There were no differences in the maternal occupational status among third-wave participants compared with those in the first wave: the majority of the mothers had the highest or second highest occupational background (Table 2).

## What has been measured?

The participants filled in an online survey that covered a wide range of topics. The new measures are presented below and in Table 1. The survey was available in both official languages of Finland, namely Finnish and Swedish. The participants were provided with a measuring tape and they reported their height, weight, and waist circumference (midway between the hip bones and ribs) according to detailed written and graphical instructions. BMI was calculated and categorized into thinness (<18.5 kg/m^2^), normal weight (18.5–24.9 kg/m^2^), overweight (25.0–29.9 kg/m^2^), and obesity (≥30.0 kg/m^2^). As the anthropometry was self-assessed and self-reported, we investigated the distributions and centroid of height, weight, and waist by using the Mahalanobis distance (Supplementary Figure S2). In total, 39 outliers were identified. These were manually evaluated, resulting in the removal of 16 discordant anthropometric measurements.

Detailed information about eating habits and dietary intakes was collected by using a 134-item food frequency questionnaire that covered the whole diet. The questionnaire was developed and validated by THL [16–18]. The daily food consumption and nutrient intake will be calculated by utilizing the Finnish food composition database Fineli [20].

New questions on pets were developed by the Folkhälsan Research Center. As the data collection was conducted partly during the COVID-19 pandemic, we also added questions concerning changes in lifestyle factors and mood due to restrictions caused by the pandemic.

## What has it found? Key findings and publications

Our key finding concerns the weight status of young adults. The mean BMI was 22.9 (3.5) kg/m^2^, which did not differ between genders. Approximately 73% of participants were of normal weight, while the combined frequency of participants with overweight and obesity was 22% (Table 2).

As the cohort was established to follow changes in the weight status of the participants, a longitudinal analysis was conducted. Information on BMI at both the first and third waves was available from 3557 participants (Table 3). In total, 2143 participants out of 2693 (80%) maintained normal weight, while 303 out of 436 participants (69%) were still living with overweight or obesity after the 8-year follow-up. The number of participants who were living with overweight or obesity increased from 436 (12%) to 770 (22%) among the 3557 participants. Thus, 467 participants (13%) transitioned from having normal weight or thinness to living with overweight or obesity during the follow-up, while 133 (4%) who were initially living with overweight/obesity achieved normal weight. The prevalence of central obesity, defined by a waist-to-height ratio of ≥0.5, increased from 9% to 16% during the same period.

**Table 3.** Weight status based on BMI at the first (mean age 11.2 years) and third (mean age 19.5 years) wave. For participants aged <18 years, sex- and age-specific BMI cutoffs by the International Obesity Task Force [41] were used to categorize weight status.

		Weight status (third wave)									
		Thinness		Normal weight		Overweight		Obesity		Total (first wave)	
		*n*	%	*n*	%	*n*	%	*n*	%	*n*	%
**Weight status (first wave)**	**Thinness**	98	2.8	324	9.1	6	0.2	0	0.0	428	12.0
	**Normal weight**	89	2.5	2143	60.2	398	11.2	63	1.8	2693	75.7
	**Overweight**	0	0.0	126	3.5	158	4.4	88	2.5	372	10.5
	**Obesity**	0	0.0	7	0.2	20	0.6	37	1.0	64	1.8
**Total (third wave)**		187	5.3	2600	73.1	582	16.4	188	5.3	3557	100

Regarding substance use, 3.2% reported that they smoked regularly, 0.4% and 5.0% used electronic cigarettes and snuff regularly, respectively, and 83.7% consumed alcohol. The use of these substances has an age limit of 18 years in Finland.

So far, we have produced ∼40 peer-reviewed articles and trained four PhD graduates by using the Fin-HIT data and material that focus on obesity, saliva microbiota, digital media use, sweet treat consumption, and autoimmune diseases (https://www.finhit.fi/publications). We have planned the following publications that utilize the third-wave data in combination with previously collected data:

“Association of chronotype/circadian misalignment with weight trajectory and mental health in young adults”: circadian preference or chronotype refers to an individual’s tendency to be active at different intervals during the day. The tendency to be an evening type has been associated with several unfavorable health behaviors and outcomes in adulthood, such as unhealthy eating, excess weight, impaired cardiometabolic health, and elevated risk of depression [21, 22]. We will study the associations of chronotype and circadian misalignment with weight trajectories throughout school age and with mental health indicators at emerging adulthood.

“Childhood overeating and food consumption”: overeating—that is, eating beyond one’s nutritional need—is a complex physiological and psychological process that is influenced by genetics, environment, individual behavior, and neural processes [23, 24], and greatly affects weight development. Our cross-sectional data show that the consumption frequency of sweet treats, fruits and vegetables, and fast food at the age of 11 years did not differ between children with and without a parent-reported tendency towards overeating. Overall, data on food consumption that are related to overeating in young adults remain sparse. We aim to investigate whether overeating or a genetic predisposition to it affects specific food consumption and dietary quality in emerging adulthood.

“Exploring the associations between mental health and weight development”: obesity and poor mental health frequently co-occur in adulthood [25], but their co-development during adolescence and young adulthood has remained unclear. Mental distress, such as symptoms of depression and anxiety, has previously been associated with higher odds of obesity [26] whereas positive aspects of mental health such as high self-esteem have been associated with lower odds of obesity [27]. As a possibility for reverse causality exists, we aim to investigate both the risk and protective factors for mental health, and the associations between mental health and weight development, by using longitudinal study designs and considering genetic predisposition.

“The impact of pet ownership and support from pets on young adults’ mental health during the COVID-19 pandemic”: the lockdown due to the COVID-19 pandemic uniquely affected young adults worldwide during an important period for social development. During this time, companion animals potentially played a role in providing emotional support to families [28]. We will investigate in a case–control setting the contribution of pet ownership to young adults’ mental health and mood during lockdown in spring 2021. By considering different aspects of the human–animal bond, such as time spent with the pet and the perceived support provided by the pet, we aim to clarify the potential mental health benefits that pets may offer to young adults during crises such as global pandemics.

## What are the main strengths and weaknesses?

The Fin-HIT cohort is a longitudinal study with a large sample size and data collected at three time points throughout adolescence and early adulthood. The research data comprise anthropometric measures, questionnaire data, and biological samples that have been used for multi-omics purposes. Currently, we have genome-wide genetic variables with SISu v3 imputation available from ∼4000 participants, saliva microbiota with 16S profiling from 1500 participants, saliva metabolome with untargeted UHPLC-HRMS metabolomic fingerprinting [29] from 500, and concentration of salivary matrix metalloproteinase-8 from 450 repeated samples. The linkage to national health registers that include disease diagnoses (ICD-10 codes), oral health indices, weight trajectories throughout the school age, and purchased medicines (Anatomical Therapeutic Chemical codes) expands the insight into participants’ health. In addition, we have demographic and behavioral factors available for 6000 parents, allowing investigations into parent–child pairs and trans-generation effects.

The participation rate in the third wave was 34.5% of the initial cohort, which is lower than in the second wave (>50%). Our questionnaire was extensive, with multiple scales divided into six sections, making it time-consuming to complete, which could have partly contributed to the lower overall participation rate. However, we had an automated e-mail reminder system for participants who had started filling in the questionnaire that prompted them to conclude their pending tasks, hopefully increasing the completion rate of the survey. Fortunately, we were able to reconnect with some of the participants who had participated in the first wave but did not participate in the second wave, as shown in Figure 2A. Generally, individuals in emerging adulthood are considered challenging to recruit, engage, and retain in research [4]. To increase our response rate and retain our participants, we used several endorsement methods such as branding, spokespeople, incentives such as a free audiobook or e-book and rental movie, and reminders. In addition, we provided feedback on their sleep duration, physical activity, alcohol use, and symptoms of depression. Our analysis showed that the background characteristics of adhering participants such as distributions of current weight status, parental sex, and maternal occupational status remained relatively stable between the first and third waves. However, participants’ sex distribution changed, with the rate of young men dropping from 47.6% to 38.5%, which is a clear limitation. Typically, between ages 18 and 25 years, men undergo obligatory military service and, generally, 75% of the men of every age class attend it [30]. Constraint caused by military service may explain the drop in male attendance in the third wave but, in general, it seems that low participation in health surveys is especially typical of young men [31, 32].

## Can I get hold of the data? Where can I find out more?

Enquiries for collaboration and data access can be sent to Principal Investigator Heli Viljakainen (heli.viljakainen@helsinki.fi, phone +358 50 4485660). Fin-HIT saliva samples with selected questionnaire data are deposited in the THL Biobank (https://thl.fi/en/research-and-development/thl-biobank/for-researchers/sample-collections/fin-hit-study) where interested researchers may submit applications for data utilization (https://thl.fi/en/research-and-development/thl-biobank/for-researchers/application-process). In addition, pseudonymized research datasets are stored in the European Genome-phenome Archive, EGA (https://ega-archive.org/dacs/EGAC00001000928) with authorized access for external partners who are interested in the saliva microbiome. You can also reach us through our web pages (https://www.finhit.fi).

## Ethics approval

The Ethics Committee of the Hospital District of Helsinki and Uusimaa approved the Fin-HIT study (approval number 169/13/03/00/10) and the third-wave data collection (HUS/1772/2017, 19 February 2020). All study procedures adhered to the Declaration of Helsinki and its later amendments or followed comparable ethical standards.

## Supplementary Material

dyaf025_Supplementary_Data
