# Supplementary material for: Cohort Profile Update: Finnish Health in Teens (Fin-HIT)
Source: Int J Epidemiol. 2025 Mar 25;54(2):dyaf025. doi: 10.1093/ije/dyaf025 (PMC11938422; doi:10.1093/ije/dyaf025)
Supplement: dyaf025_Supplementary_Data [file dyaf025_supplementary_data.docx]

Supplementary Figures:


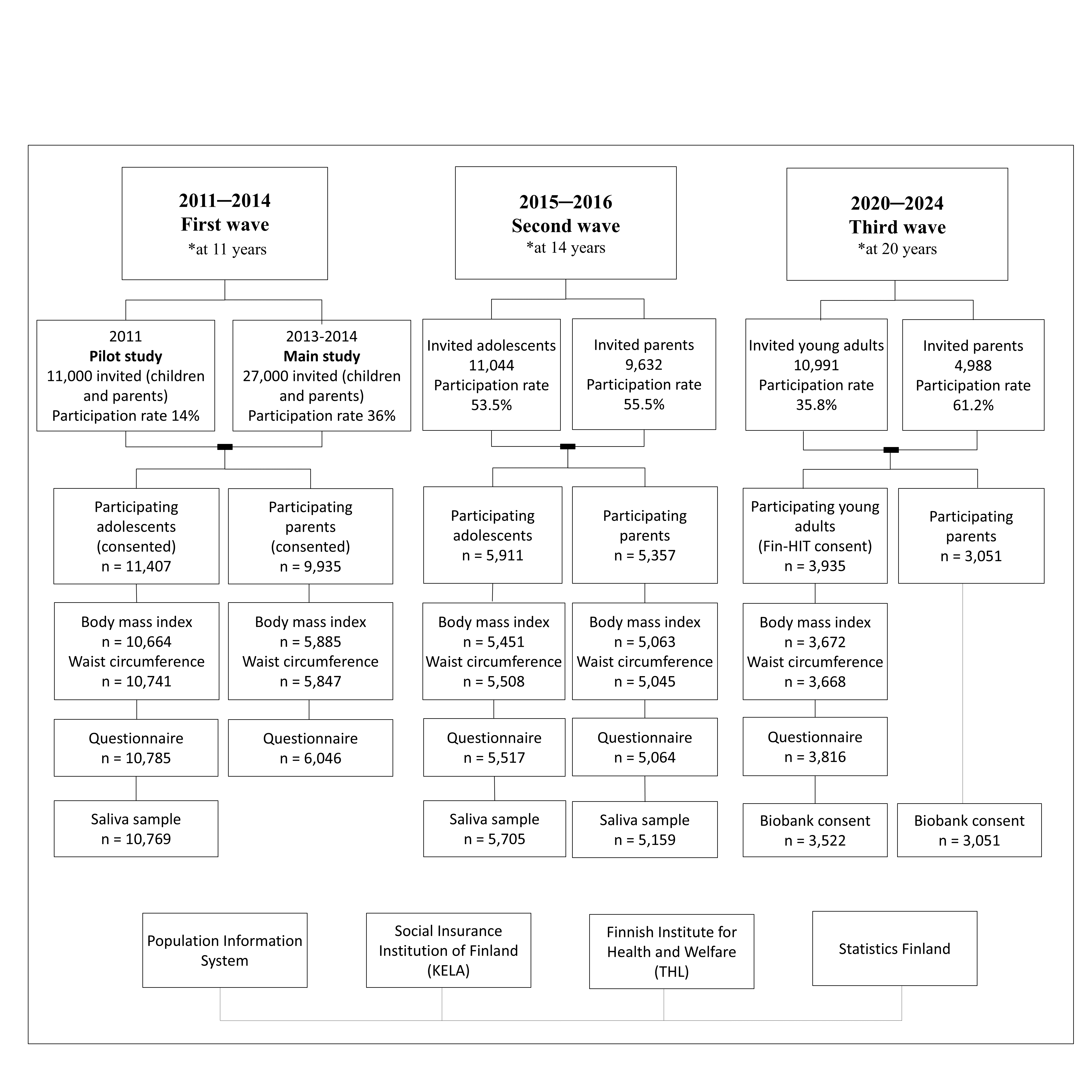


**Figure S1.** Fin-HIT cohort flow chart.

*The rounded mean age of participants.

This figure is adapted from Figueiredo et al. 2019 (1), and extended to cover the third wave data collection. Written permission was received from the copyright owner.

**
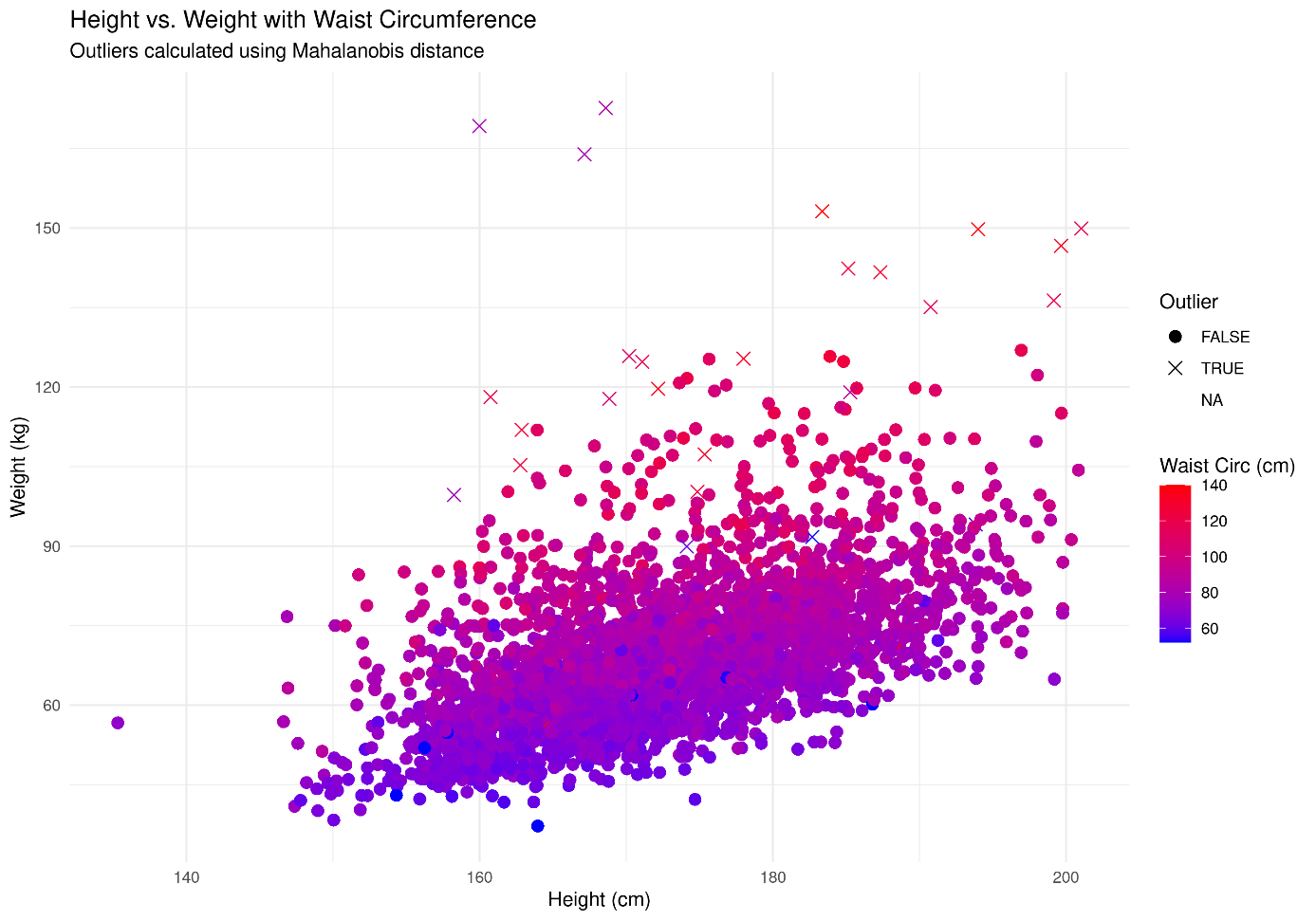
Figure S2.** Outlier recognition in anthropometric measurements at the third wave data collection by Mahalanobis plot.
